# Effect of Tai Chi on psychological disorder in college students: A protocol of systematic review and meta-analysis

**DOI:** 10.1097/MD.0000000000020409

**Published:** 2020-06-05

**Authors:** Li-Li Jiang, Yang Meng, Qian Zhang, Wei Pan

**Affiliations:** a Department of Urology; b Department of Gastroenterology, The Affiliated Hongqi Hospital of Mudanjiang Medical University, Mudanjiang, Heilongjiang, China.

**Keywords:** college students, effect, psychological disorder, Tai Chi

## Abstract

**Background:**

This study will explore the effect of Tai Chi on psychological disorder (PD) in college students (CS).

**Methods:**

A comprehensive literature search of relevant randomized controlled trials will be carried out in electronic databases from inception to the February 29, 2020: PUBMED, EMBASE, Cochrane Library, Web of Science, Chinese Biomedical Literature Database, and China National Knowledge Infrastructure. There are not limitations related to the language and publication time. Study quality will be assessed by Cochrane risk of bias tool, and evidence quality will be appraised by the grading of recommendations assessment, development, and evaluation approach. RevMan 5.3 software will be exploited to perform statistical analysis.

**Results:**

The protocol of this proposed study will investigate the effect of Tai Chi on PD in CS.

**Conclusion:**

The findings of this study will provide helpful evidence for clinical practice, and health related policy maker to develop a better intervention plan for PD in CS.

**Study registration number:**

INPLASY202040140.

## 1 Introduction

When young adults attend university, they often experience a quite long term period of psychological challenges and adaptations, because of the total different lifestyles compared with their high school.^[1–3]^ Such sudden changes are various in their habits, social and academic lives.^[4–6]^ Thus, the occurrence of psychological disorder (PD) in college students (CS) should be paid highly attention.^[7–8]^ It is reported that the prevalence of PD can be up to 50% in universities.^[9–12]^ So, it is very important to manage PD in CS.

Tai Chi (TC), an ancient Chinese healing exercise, is reported to manage a lot of disorders effectively, especially for PD (such as depression and anxiety).^[13–18]^ Several studies have assessed the effect of TC on PD in CS, yet no consensus is reached.^[19–28]^ Therefore, this study will critically assess the effect of TC on PD in CS.

## 2 Methods

### 2.1 Study registration

This study has been funded and registered on INPLASY202040140. We have reported it based on the guideline of preferred reporting items for systematic reviews and meta-analysis protocol statement.^[29]^

### 2.2 Criteria for included studies

#### 2.2.1 Study types

This proposed study will include randomized controlled trials (RCTs) alone that explore the effect of TC on PD in CS. We will eliminate other studies, such as nonclinical trials, uncontrolled trials, and non-RCTs.

#### 2.2.2 Participants

All CS who were diagnosed as PD will be included, regardless country, gender, and age.

#### 2.2.3 Interventions

The participants in the treatment group received TC. However, we will exclude studies utilized TC with other managements.

There are no limitations related to the treatments on subjects in the control group. However, studies used the combination of TC and other treatments will not be included.

#### 2.2.4 Outcomes

Primary outcomes are depression and anxiety. Both depression and anxiety were measured by any related scales as reported in the primary trials.

Secondary outcomes are stress (as assessed by perceived stress scale or other related scales), panic (as evaluated by panic disorder severity scale or other associated scales), health-related quality of life (as appraised by 36-item short form survey or other connected surveys), and incidence of adverse events.

### 2.3 Strategy of literature searches

We will perform a comprehensive literature search of relevant RCTs in electronic databases from inception to the February 29, 2020 without restrictions of language and publication time: PUBMED, EMBASE, Cochrane Library, Web of Science, Chinese Biomedical Literature Database, and China National Knowledge Infrastructure. A preliminary search strategy is developed for Cochrane Library (Table 1). Similar search strategies will be adapted for other electronic databases.

**Table 1:**
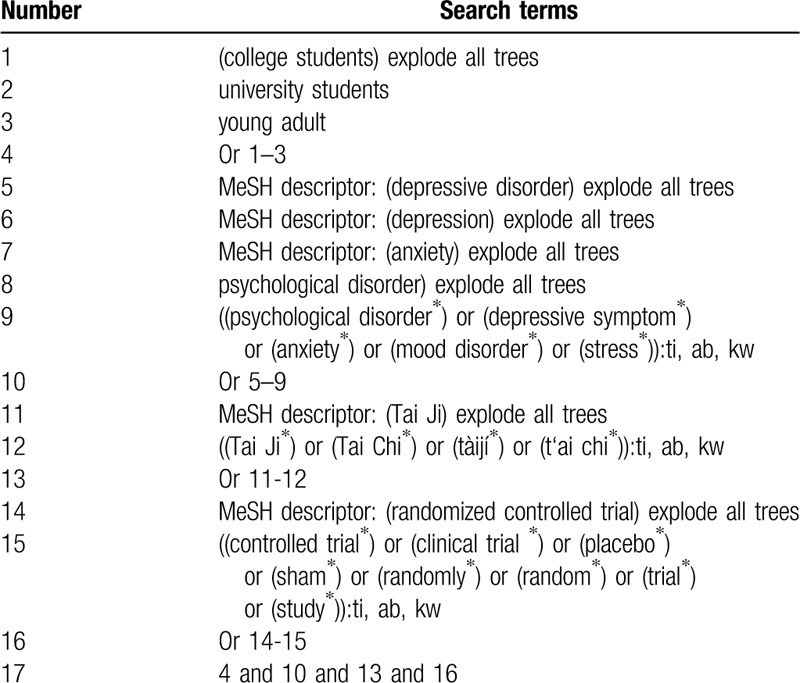
Search strategy of Cochrane Library.

Besides, we will also examine Google Scholar, ongoing trials from clinical trial registries, and reference lists of included RCTs.

### 2.4 Data collection

#### 2.4.1 Study selection

We will import all sought literatures to EndNote X7, and will eliminate all duplicates. Two investigators will scan titles/abstracts of potential studies, and all irrelevant studies will be removed. The full-text of remaining articles will be further read based on all inclusion criteria. Any different opinions will be resolved by discussion with a third investigator. The whole process of study selection is displayed in a preferred reporting items for systematic reviews and meta-analysis flow chart.

#### 2.4.2 Data extraction

For all eligible studies, 2 investigators will independently perform data extraction using a predefined data extraction sheet. It consists of general information (eg, first author, journal, country, year of publication), clinical features (eg, sample size, details of interventions, controls, outcomes, safety, and study findings), study methods (eg, trial design, randomization, blind, allocation, concealment), and other information (eg, conflict of interest, funding). Any discrepancies will be settled by a third investigator through consultation.

#### 2.4.3 Dealing with missing data

If unclear or missing data will be examined, we will contact primary trial authors to request it. If it is not available, we will analyze current data using an intention-to-treat analysis.

### 2.5 Study quality assessment

For all included trials, 2 investigators will assess study quality separately through 7 aspects, and each one is further judged as low, unclear, or high risk of bias. Any disagreements will be figured out by discussion with help of a third investigator.

### 2.6 Statistical analysis

#### 2.6.1 Data synthesis

RevMan 5.3 software will be undertaken to perform statistical analysis. All continuous values will be estimated as weighted mean difference or standardized mean difference and 95% confidence intervals, and all dichotomous values will be demonstrated as risk ratio and 95% confidence intervals. *I*^2^ test will be identified to check statistical heterogeneity. *I*^2^ ≤ 50% suggests reasonable heterogeneity, and we will use a fixed-effects model. *I*^2^ > 50% indicates remarkable heterogeneity, and we will utilize a random-effects model. A meta-analysis will be carried out based on the acceptable heterogeneity and ample data from sufficient trials. On the other hand, we will perform a subgroup analysis to examine heterogeneity sources.

#### 2.6.2 Subgroup analysis

A subgroup analysis will be carried out according to the different types of treatments, comparators, and outcome measurements.

#### 2.6.3 Sensitivity analysis

A sensitivity analysis will be performed to test the robustness of study results by taking away low quality trials.

#### 2.6.4 Reporting bias

A funnel plot^[30]^ and Egger regression test^[31]^ will be examined to find out any possible reporting bias when more than 10 trials are included.

### 2.7 Ethics and dissemination

All data collected in this study were from published trials, thus, no ethic approval is required. This study will be published on a peer-reviewed journal.

## 3 Discussion

Presently, no systematic review investigates the effect of TC on PD in CS, although an increasing number related studies have been published. Thus, it is necessary to evaluate already published studies of this topic in this domain. The findings of this study will provide a systematic and comprehensive synthesis of current published data to assess the effect of TC on PD in CS. It will also provide helpful evidence for clinical practice and patients, as well health related policy makers.

## Author contributions

**Conceptualization:** Li-Li Jiang, Qian Zhang, Wei Pan.

**Data curation:** Li-Li Jiang, Yang Meng, Wei Pan.

**Formal analysis:** Li-Li Jiang, Yang Meng, Qian Zhang, Wei Pan.

**Investigation:** Wei Pan.

**Methodology:** Li-Li Jiang, Yang Meng, Qian Zhang.

**Project administration:** Wei Pan.

**Resources:** Li-Li Jiang, Yang Meng, Qian Zhang.

**Software:** Li-Li Jiang, Yang Meng, Qian Zhang.

**Supervision:** Wei Pan.

**Validation:** Li-Li Jiang, Qian Zhang, Wei Pan.

**Visualization:** Li-Li Jiang, Yang Meng, Wei Pan.

**Writing – original draft:** Li-Li Jiang, Qian Zhang, Wei Pan.

**Writing – review and editing:** Li-Li Jiang, Yang Meng, Wei Pan.
